# TOR signalling is required for host lipid metabolic remodelling and survival following enteric infection in *Drosophila*

**DOI:** 10.1242/dmm.049551

**Published:** 2022-05-09

**Authors:** Rujuta Deshpande, Byoungchun Lee, Yuemeng Qiao, Savraj S. Grewal

**Affiliations:** Clark H Smith Brain Tumour Centre, Arnie Charbonneau Cancer Institute, Alberta Children's Hospital Research Institute and Department of Biochemistry and Molecular Biology Calgary, University of Calgary, Alberta T2N 4N1, Canada

**Keywords:** *Drosophila*, TOR, Infection, Lipid metabolism, Physiology

## Abstract

When infected by enteric pathogenic bacteria, animals need to initiate local and whole-body defence strategies. Although most attention has focused on the role of innate immune anti-bacterial responses, less is known about how changes in host metabolism contribute to host defence. Using *Drosophila* as a model system, we identify induction of intestinal target-of-rapamycin (TOR) kinase signalling as a key adaptive metabolic response to enteric infection. We find that enteric infection induces both local and systemic induction of TOR independently of the Immune deficiency (IMD) innate immune pathway, and we see that TOR functions together with IMD signalling to promote infection survival. These protective effects of TOR signalling are associated with remodelling of host lipid metabolism. Thus, we see that TOR is required to limit excessive infection-mediated wasting of host lipid stores by promoting an increase in the levels of gut- and fat body-expressed lipid synthesis genes. Our data support a model in which induction of TOR represents a host tolerance response to counteract infection-mediated lipid wasting in order to promote survival.

This article has an associated First Person interview with the first author of the paper.

## Abstract (precis)

**Summary:** Enteric infection in *Drosophila* leads to increased TOR signalling, which is required to prevent excess wasting of fat stores and promote survival.

## INTRODUCTION

A central role for the immune system is to sense invading bacterial pathogens and then trigger appropriate defence responses. One defence strategy is to decrease pathogen load (Schneider and Ayres, 2008). Central to this mechanism are the innate immune responses. These are responsible for sensing invading bacteria at the sites of infection and then activating both local and whole-body host anti-bacterial responses (Buchon et al., 2014). It is also becoming clear that changes in host metabolism are another important defence strategy against infection (Ayres and Schneider, 2012; Medzhitov et al., 2012; Troha and Ayres, 2020). The innate immune response can be energetically costly, and these metabolic changes are often needed to fuel the immune response (Krejcova et al., 2019; Man et al., 2017). In addition, metabolic reprogramming is often essential for animals to adapt to and tolerate the presence of pathogens (Ganeshan et al., 2019; Sanchez et al., 2018; Wang et al., 2018, 2016; Weis et al., 2017). Indeed, there is increasing appreciation that, for a given pathogen load, inter-individual differences in survival outcomes are determined by differences in the ability to mediate appropriate metabolic adaptations to infection (Troha and Ayres, 2020). However, compared with our understanding of innate immunity, less is known about how these metabolic adaptations promote host fitness upon infection.

*Drosophila* has provided a powerful model system to study host defence responses to enteric bacterial infection (Buchon et al., 2014; Lee and Lee, 2018). Upon ingestion of pathogenic bacteria, the intestine triggers two main responses to mount antibacterial defences. The first involves activation of the conserved Immune deficiency (IMD)/Relish/NF-κB pathway by Gram-negative bacteria, which leads to production of antimicrobial peptides (AMPs) (Buchon et al., 2014). The second involves bacteria-derived uracil, which stimulates reactive oxygen species (ROS) production in intestinal epithelial cells (Lee et al., 2018, 2015). Both pathways can promote local antimicrobial responses in the intestine and also trigger signalling from the intestine to other tissues to promote a whole-body anti-bacterial response such as production of AMPs from the fat body (Wu et al., 2012; Yang et al., 2019). Enteric infection can also alter both intestinal and whole-body metabolism, but the contributions of these effects to defence against pathogens are not fully clear (Galenza and Foley, 2019; Lee and Lee, 2018; Wong et al., 2016).

Target-of-rapamycin (TOR) kinase is a conserved regulator of cell, tissue and whole-body metabolism (Ben-Sahra and Manning, 2017; Howell et al., 2013; Saxton and Sabatini, 2017). In general, TOR is activated under favourable conditions (e.g. growth factor stimulation and nutrient availability) to stimulate cellular anabolic metabolism and promote growth. In contrast, under stress conditions such as starvation, hypoxia or oxidative damage, TOR is inhibited to promote catabolic metabolism to ensure cell survival. The utility of *Drosophila* genetics has also been instrumental in showing how TOR activation in specific tissues can trigger and coordinate whole-body-level physiological and metabolic responses (Boulan et al., 2015; Grewal, 2009; Texada et al., 2020). These effects rely on the ability of TOR signalling to promote inter-organ communication and endocrine signalling, and have been shown to be essential for organismal responses to environmental changes such as altered nutrition and hypoxia (Boulan et al., 2015; Koyama et al., 2020).

Given the central role for TOR in controlling whole-body physiology and metabolism, some studies have begun to explore its role in responses to bacterial infection in *Drosophila*. However, these studies have differed in their conclusions about whether TOR activity is helpful or harmful to host immune responses and fitness upon infection. In some cases, it was reported that reduced TOR activity provided a benefit to the host. For example, enteric infection with *Erwinia carotovora carotovora*, a Gram-negative bacterium, was shown to inhibit TOR and lead to increased lipid breakdown in the gut (Lee et al., 2018). This loss-of-TOR-mediated metabolic shift to lipid catabolism was required for the antimicrobial ROS response and increased the host resistance to enteric infection. Similarly, another report showed that lowered TOR activity upon enteric infection could induce AMPs (Varma et al., 2014). Finally, one report showed that lowering TOR activity, either genetically or by nutrient restriction, was sufficient to increase survival upon systemic infection with either *Pseudomonas aeruginosa* or *Staphylococcus aureus* (Lee et al., 2017*)*. In contrast to these findings, other studies showed that lowered TOR activity is detrimental to hosts upon infection. For example, enteric infection with *Pseudomonas entomophila* decreased gut TOR activity, leading to suppressed intestinal protein synthesis, which reduced immune responses and prevented proper intestinal tissue repair (Chakrabarti et al., 2012). Another study also showed that TOR inhibition reduced fly survival upon systemic infection with *Burkholderia cepacia* (Allen et al., 2016). The reasons for these differences in the links between TOR and infection response in *Drosophila* may be due to the different bacterial infections used or because of differences in host metabolic or nutrient status. Nevertheless, they indicate that further work is required to clarify how TOR may play a role in immune and metabolic responses to infection. We address this issue in this paper. We show that enteric infection leads to increased TOR signalling independently of innate signalling, and that this induction is required to remodel host lipid metabolism and promote survival.

## RESULTS

### Enteric bacterial infection stimulates local and systemic TOR signalling

**Fig. 1.**
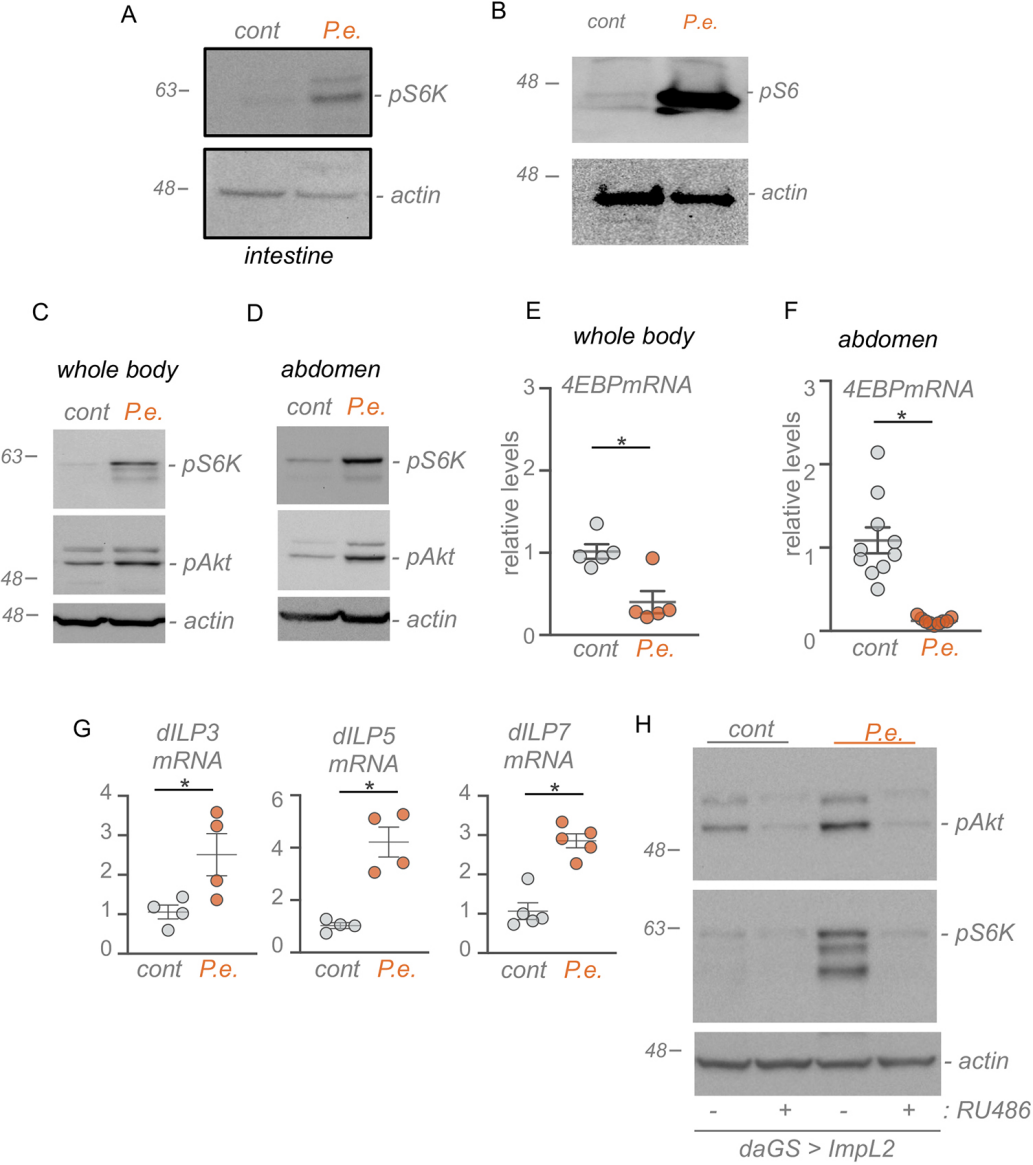
**Enteric bacterial infection stimulates local and systemic TOR activity.** (A,B) Western blots of intestines from adult flies subjected to 4 h oral *P. entomophila* (*P.e.*) infection using antibodies to phosphorylated S6K (pS6K) (A), phosphorylated S6 (pS6) (B) and actin (as a loading control). (C,D) Western blots of either whole animals (C) or isolated abdominal samples (D) from adult flies subjected to 4 h oral *P. entomophila* infection using antibodies to phosphorylated S6K (pS6K), phosphorylated Akt (pAkt) and actin (as a loading control). (E,F) qPCR analysis of the FOXO target gene, *4EBP*, from either whole-body samples (E) or abdominal samples (F) of control or *P. entomophila*-infected flies. Bars represent mean±s.e.m., individual data points are plotted as circles. (G) qPCR analysis of ILP mRNAs from whole-body samples of control or *P. entomophila-*infected flies. (H) Western blots of whole-body samples from control versus *P. entomophila*-infected adult flies (genotype: *daGAl4 GeneSwitch/+; UAS-ImpL2/+*) using antibodies to phosphorylated S6K (pS6K), phosphorylated Akt (pAkt) and actin (as a loading control). ImpL2 induction was achieved by feeding flies RU486 for 3 days before infection (+ RU486). *P<0.05, two-tailed unpaired Student's *t*-test.

TOR kinase couples environmental signals to changes in cellular metabolism. Generally, TOR has been shown to be activated by favourable conditions (e.g. abundance of nutrients and growth factors) and inhibited by stress conditions (e.g. starvation, low oxygen, oxidative stress). We were interested in examining how TOR activity might be affected by enteric bacterial infection. We first infected flies with the Gram-negative bacteria *P. entomophila* for 4 h and then dissected intestines for western blotting. At this time point following feeding, *P. entomophila* is able to colonize fly intestines as previously described (Fig. S1A). Ribosomal protein S6 kinase (S6K) is directly phosphorylated and activated by TOR, hence we used western blotting for phosphorylated S6K as a readout for TOR activity. We found that oral *P. entomophila* feeding led to increased phosphorylated S6K levels (Fig. 1A). This increase was blocked by pre-feeding the flies rapamycin, a TOR inhibitor, indicating that the induction of phosphorylated S6K was through an increase in TOR activity (Fig. S1B). We also examined phosphorylation of ribosomal protein S6, a downstream target of ribosomal protein S6 kinase, and we saw that 4 h of oral *P. entomophila* infection also induced phosphorylated S6 levels in the intestine (Fig. 1B). When we performed immunostaining with the anti-phosphorylated S6 antibody, we also saw an increase in TOR activity particularly in the anterior region of the midgut (Fig. S1C). The increased phosphorylated S6 staining was seen in GFP-marked stem cells/embryoid body (EB) cells, consistent with a previous report that showed that enteric infection induces TOR signalling in intestinal stem cells (Haller et al., 2017). However, we also saw phosphorylated S6 staining in the surrounding large polyploid GFP-negative cells, which are likely the enterocyte epithelial cells (Fig. S1D). A conserved function of TOR is the stimulation of cellular protein synthetic capacity, in large part mediated via upregulation of tRNA and rRNAs, and genes involved in ribosome synthesis (Ghosh et al., 2014; Killip and Grewal, 2012; Marshall et al., 2012; Mayer and Grummt, 2006; Rideout et al., 2012). When we used quantitative real time polymerase chain reaction (qRT-PCR) to measure RNA levels in intestinal samples, we saw that oral *P. entomophila* infection led to an increase in tRNA and pre-rRNA levels and an increase in mRNA levels of three ribosome biogenesis genes, *Nop5*, *ppan* and *Fibrillarin*, upon oral *P. entomophila* infection (Fig. S1E). We explored the effects of oral bacterial infection further by performing a time course following oral *P. entomophila* feeding. We saw that the induction of TOR was rapid (within 4 h of infection) and persisted for 24 h of the oral infection period (Fig. S2A). We also found that this induction of TOR was similar in males and females (Fig. S2B). Moreover, the effects of *P. entomophila* appear to be limited to adults, as 4 h oral infection in larvae did not increase phosphorylated S6K levels, and in fact showed a small decrease (Fig. S2C). We also tested two other pathogenic Gram-negative bacteria, *Vibrio cholera* and *E. carotovora carotovora*. We again used western blotting for phosphorylated S6K to measure TOR and saw that oral infection with *V. cholera* and *E. carotovora carotovora* both led to increased intestinal TOR activity (Fig. S2D). Together, these data indicate that induction of intestinal TOR kinase signalling is a rapid response to enteric Gram-negative bacterial infection.

As well as affecting intestinal physiology, enteric infection can also trigger non-autonomous, systemic responses that can impact physiology in remote tissues. We therefore examined whether enteric infection might induce TOR activity more widely. We found that oral *P. entomophila* feeding led to increased phosphorylated S6K levels in both whole animals and in isolated abdomens, which are enriched in fat body, a tissue in which TOR plays important roles in the control of organismal metabolism and physiology (Fig. 1C,D). These results suggest that enteric bacterial infection can lead to non-autonomous induction of TOR in other metabolically important tissues. One main way that systemic TOR activity can be controlled is through the endocrine insulin signalling pathway. In *Drosophila*, seven insulin-like peptides (ILPs) are secreted into the haemolymph from the brain and other tissues where they can function in a long-range manner to activate a conserved PI3K/Akt kinase signalling pathway in target tissues. Activated Akt can signal by stimulating TOR or by blocking the nuclear localization and activity of the FOXO transcription factor. We therefore explored whether enteric infection might mediate non-autonomous induction of TOR signalling by upregulating systemic insulin signalling. We first measured phosphorylation of Akt as a read-out of insulin signalling. We found that, following oral infection with *P. entomophila*, there was an increase in Akt phosphorylation in both whole-animal sample and isolated abdominal tissue samples (Fig. 1C,D; Fig. S3B,C), suggesting an increase in systemic insulin signalling. Interestingly, this increase in Akt phosphorylation was not seen in the intestine following oral infection with *P. entomophila* (Fig. S3A). We also measured mRNA levels of *4EBP* (also known as *Thor*), a FOXO target gene. We found that oral *P. entomophila* infection was sufficient to reduce *4EBP* levels in both whole-animal and isolated abdominal samples (Fig. 1E,F), which is also consistent with elevated insulin signalling. One main way that insulin signalling can be induced is through increased production of the ILPs. We found that *P. entomophila* infection was sufficient to increase expression of three ILPs (ILP3, 5 and 7) (Fig. 1G). To test whether this increase in insulin signalling could explain the induction of systemic TOR signalling upon infection, we examined the effects of overexpression of ImpL2, the *Drosophila* homolog of the mammalian insulin-like growth factor binding protein. ImpL2 binds to ILPs and inhibits their ability to signal through the insulin receptor. We found that ubiquitous overexpression of ImpL2 was sufficient to block insulin signalling (as measured by Akt phosphorylation) (Fig. 1H) and to prevent that infection-mediated increase in TOR signalling, as measured by S6K phosphorylation (Fig. 1H). Together, these data suggest that enteric infection can induce direct stimulation of intestinal TOR activity and also a non-autonomous upregulation of whole-body insulin/TOR signalling.

We next explored whether other enteric intestine stresses might also regulate TOR signalling. Feeding flies with three known chemical intestine stressors, bleomycin (a DNA damaging agent), dextran sodium sulphate (DSS, a detergent) and paraquat (an oxidative stressor), had no effect on intestinal TOR activity (Fig. S4A). We also explored two nutrient stresses – high sugar and high fat. However, we saw that feeding the flies either a high sugar (40% sucrose) or high fat (30% lard) supplemented diet also did not have any effect on TOR signalling in the intestine (Fig. S4B). Together, our data suggest that the induction of TOR appears to be specific to oral bacterial infection. Interestingly this induction occurs in a bacterial concentration-manner and is seen by low levels of bacterial infection (Fig. S4C).

### Infection-mediated TOR stimulation is independent of IMD and ROS signalling

**Fig. 2.**
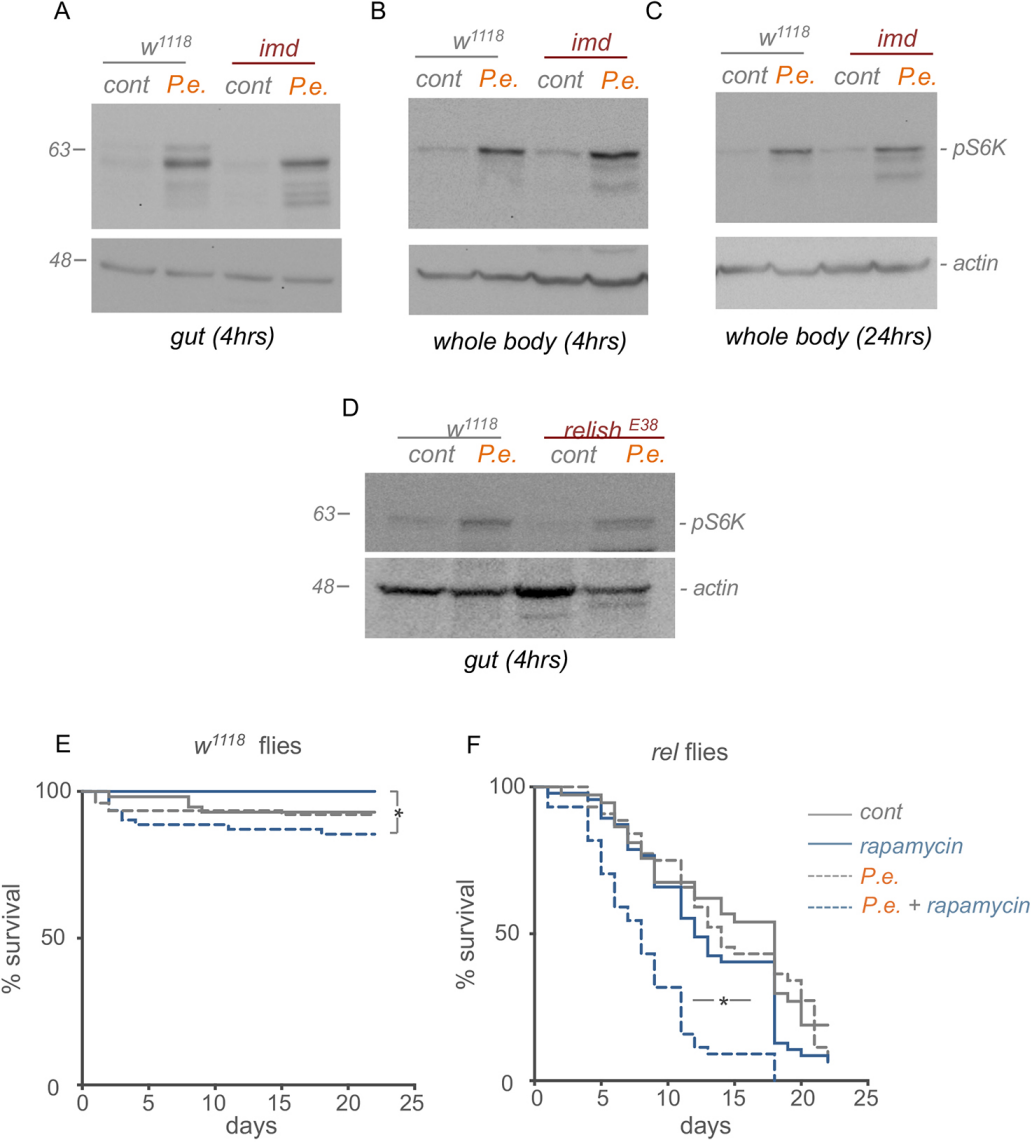
**TOR and IMD signalling function in parallel to control survival in response to enteric infection.** (A-C) Western blots of intestinal (A) or whole-body samples (B, 4 h; C, 24 h) from control versus *P. entomophila* (*P.e.*)-infected adult *w^1118^* or *imd* mutants using antibodies to phosphorylated S6K and actin (as a loading control). (D) Western blots of intestinal samples from control versus *P. entomophila*-infected adult *w^1118^* or *Relish* mutants using antibodies to phosphorylated S6K and actin (as a loading control). (E,F) Survival plot of control *w^1118^* (E) and *Relish^E20^* (*rel*) mutant (F) mated female flies subjected to 48 h oral *P. entomophila* infection. Animals were then returned to standard food and the percentage of animals surviving was counted. *N*≥50 animals per experimental condition. *P<0.05, log rank test.

A primary and well-studied response to enteric Gram-negative bacterial infection is activation of the IMD/NF-κB pathway (Kleino and Silverman, 2014). We therefore examined whether either increased IMD signalling might be the trigger for TOR induction upon oral *P. entomophila* infection. We began by examining mutants for *imd*, a death domain-containing protein that is a key effector of the IMD signalling cascade. We infected either control (*w^1118^*) or *imd* mutants with *P. entomophila* and saw that the induction of AMPs was completely suppressed in the *imd* mutants, confirming that they are impaired in proper immune signalling (Fig. S5). However, when we examined TOR activity by measuring S6K phosphorylation by western blot, we found that infection-induced increase in TOR activity both in the intestine and whole animal was unaffected in *imd* mutants (Fig. 2A-C). We also examined mutants for the NF-κB-like transcription factor, Relish, which is the downstream transcriptional effector of the IMD pathway. Again, we found that the induction of intestinal TOR upon oral *P. entomophila* infection was still observed in the *Relish* mutants (Fig. 2D). These results suggest that TOR induction seen upon enteric *P. entomophila* infection is independent of IMD signalling.

Induction of ROS signalling by bacteria-derived uracil signalling also mediates immune responses upon enteric infection with Gram-negative bacteria (Lee et al., 2015; Wu et al., 2012). However, we found that feeding uracil had no effect on TOR signalling and we also found that feeding the flies N-acetylcysteine, a potent antioxidant, along with oral *P. entomophila* feeding did not reverse the induction of TOR upon infection (Fig. S6A,B). Together with our result with paraquat, a stimulator of ROS (Fig. S4A), our data suggest that TOR induction is independent of ROS.

### Inhibiting TOR and IMD pathways simultaneously reduces survival upon *P. entomophila* infection

We next examined the consequences of TOR induction upon *P. entomophila* infection. We first examined effects on survival following enteric *P. entomophila* infection. Under our laboratory fly culture conditions, the strain of *P. entomophila* we use is not strongly pathogenic. Thus, when we infected control (*w^1118^*) adult flies for 2 days and then monitored their survival over ∼3 weeks, we saw little effect on viability compared with uninfected flies (Fig. 2E). When we infected flies and simultaneously inhibited TOR by feeding flies rapamycin, we found that this induced a slight, but significant, decrease in survival compared with flies fed rapamycin alone (Fig. 2E). We next tested the possibility that TOR functions in parallel IMD/Relish signalling to promote infection survival. We found that *Relish* mutants had a generally reduced lifespan on our normal lab food compared with control (*w^1118^*) adults (Fig. 2F). Either enteric infection with *P. entomophila* or blocking TOR with rapamycin had no effect on viability in the *Relish* mutants alone. However, when we infected *Relish* mutants and simultaneously fed them rapamycin to inhibit TOR, we saw a significant decrease in survival compared with *Relish* mutant flies subjected to infection or rapamycin treatment alone (Fig. 2F). These results suggest that, in order to survive enteric infection, an animal needs cooperative activation of both IMD/Relish and TOR signalling.

**Fig. 3.**
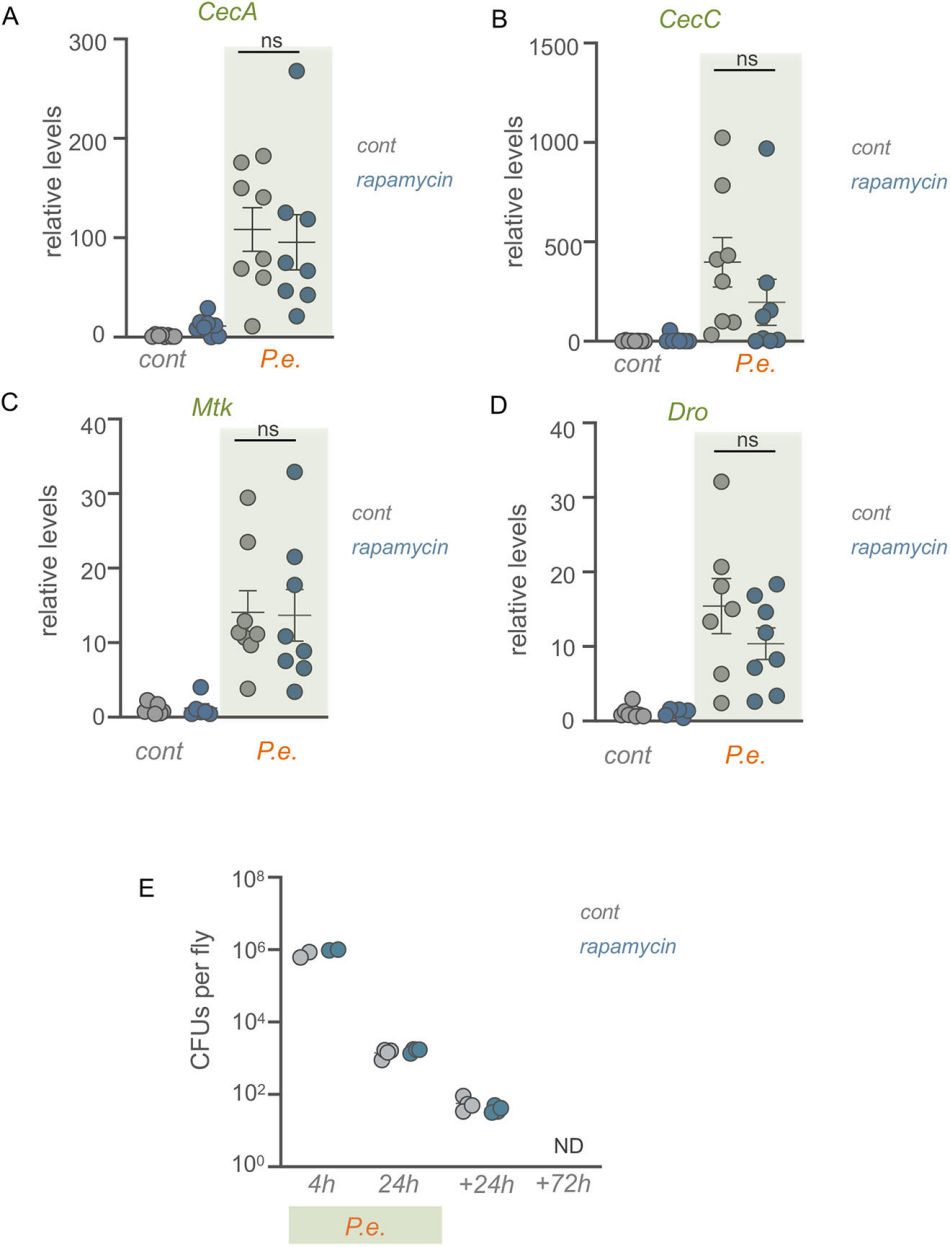
**Induction of intestinal TOR signalling is not required for systemic AMP induction.** (A-D) qRT-PCR analysis on adult *w^1118^* mated females subjected to a 24 h pre-treatment of rapamycin or DMSO control followed by 24 h oral *P. entomophila* (*P.e.*) feeding along with rapamycin. mRNA transcript levels of anti-microbial peptides (AMPs) are presented as relative changes versus control (corrected for RpS9). (E) Pathogen abundance [in colony forming units (CFUs) per fly] control and rapamycin-treated flies at 4 h and 24 h during the 2 h infection period and at 24 h and 72 h post-infection. The bars represent the mean for each condition, with error bars representing the s.e.m. and individual values plotted as symbols. Two-way ANOVA followed by two-tailed unpaired Student's *t*-test. ND, no detectable colonies; ns, not significant.

A primary infection response induced by the IMD pathway in flies is the production of AMPs (Kleino and Silverman, 2014). We saw that oral infection with *P. entomophila* led to a strong induction of several AMPs, including *Cecropin A1* and *A2* (collectively referred to as *CecA*), *Cecropin C* (*CecC*), *Metchnikowin* (*Mtk*), and *Drosocin* (*Dro*) (Fig. 3A-D). However, when we inhibited TOR by feeding flies rapamycin, this induction was not significantly affected (Fig. 3A-D). We also examined host bacterial load (as measured by colony forming units; CFU) during a 24 h infection period and a subsequent 3 day recovery period. We saw that the pathogen abundance was initially high following infection and then declined at each time point and was below detection at 3 days following infection (Fig. 3E). We also found that bacterial load was unaffected by inhibition of TOR signalling by rapamycin at each time point (Fig. 3E). These results suggest that the induction of TOR upon infection may not be required to induce antibacterial resistance responses, and that the requirement for TOR in infection survival may reflect a role in other immune responses.

### TOR induction limits lipid depletion and promotes lipid synthesis upon infection

**Fig. 4.**
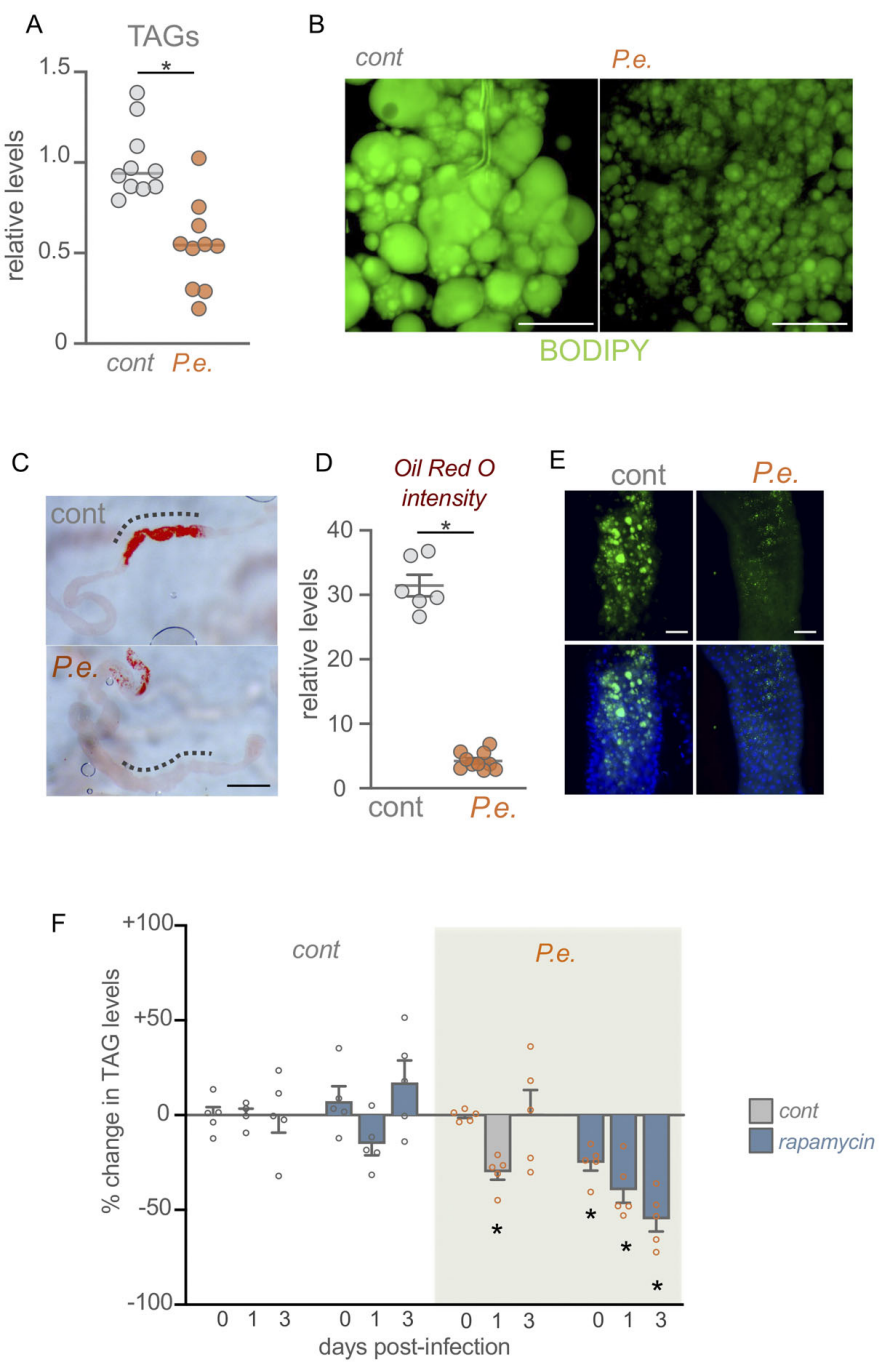
**TOR signalling is required to limit excess lipid loss following enteric infection.** (A) Total triacylglyceride (TAG) levels in control versus 48 h *P. entomophila* (*P.e.*)-infected adults. (B) BODIPY staining of fat body of *w^1118^* control and 24 h *P. entomophila*-infected mated females. Green, BODIPY. *n*=5 animals per condition. (C) Lipid droplet accumulation in the anterior region of the intestines stained with Oil- Red O (ORO) from control versus 24 h *P. entomophila*-infected flies. High levels of lipid accumulation were seen in the anterior regions of control guts (indicated with dashed line) and this was decreased in infected guts (*n*=10 per condition). (D) ORO intensities in the anterior regions (indicated with dashed lines in C) of *w^1118^* control and 24 h *P. entomophila*-infected intestines. (E) BODIPY staining of anterior regions of *w^1118^* control and 24 h *P. entomophila*-infected intestines. Green, BODIPY; blue, Hoechst DNA dye. *n*=5 guts per condition. A representative image is shown. (F) Adult *w^1118^* mated females subjected to 24 h pre-treatment of rapamycin followed by 24 h oral *P. entomophila* feeding along with rapamycin. TAG assays were performed on flies at 0, 1 or 3 days after infection. The bars represent percentage change in TAG levels (compared with uninfected control animals), normalized to the protein content for each condition. Data are mean±s.e.m., individual data points are plotted as circles. *P<0.05 (experimental group compared with the control group at the same time point), two-tailed unpaired Student's *t*-test. Scale bars: 50 µm (B,E); 200 µm (C).

Initiating immune responses against infection can be energetically costly for infected hosts. Remodelling of host metabolism is therefore increasingly recognized as an important component of immune responses (Troha and Ayres, 2020). Given that TOR kinase is a conserved regulator of metabolism, we investigated whether it might play a role in modulating host metabolic responses to infection. Lipid stores are an important metabolic fuel source. Lipids can be synthesized and stored as triacylglycerides (TAGs) in lipid droplets in the fly fat body, oenocytes and intestine. They can be then mobilized, transported to tissues and used to fuel metabolism, particularly in stress conditions (Heier and Kuhnlein, 2018). We tested for changes in TAGs upon oral *P. entomophila* infection and found a significant decrease in infected *w^1118^* adult females compared with control flies (Fig. 4A), as has been reported following systemic infection in flies (Chambers et al., 2012; Dionne et al., 2006). As the majority of the lipid stores are stored in the fat body (Zhao and Karpac, 2020), we dissected the fat bodies of infected females and found a decrease in the lipid droplet size by BODIPY staining (Fig. 4B). We also saw that enterocytes in the anterior region of the midgut accumulated lipid droplets as visualized by Oil Red O and BODIPY staining (Fig. 4C-E), as has been reported previously (Kamareddine et al., 2018a; Luis et al., 2016; Miguel-Aliaga et al., 2018). We found that oral infection with *P. entomophila* led to a depletion of these intestinal lipids (Fig. 4C-E). These results suggest that infection leads to mobilization of fat body and intestinal lipid stores, perhaps as a way to provide lipids to other tissues to fuel their metabolism, as has been recently reported (Zhao and Karpac, 2021). In support of this idea, we also saw that enteric infection increased whole-body expression of two lipid-binding proteins, apoLpp and Mtp, that are highly expressed in the fat body and that are needed for transport of lipids through the haemolymph (Fig. S7).

We next examined what role TOR might play in these lipid effects by measuring TAG levels at 0, 1 and 3 days following infection in control versus rapamycin-treated flies. In control flies, infection led to a transient decrease in TAG levels at the 1 day time point, but then TAGs recovered to the same level as uninfected flies at 3 days (Fig. 4F). Rapamycin treatment alone had no significant effect on TAG levels at any time point compared with uninfected control flies (Fig. 4F). However, when we infected flies and simultaneously fed them rapamycin to inhibit TOR, we saw a progressive depletion of TAG stores at each time point following infection (Fig. 4F).

**Fig. 5.**
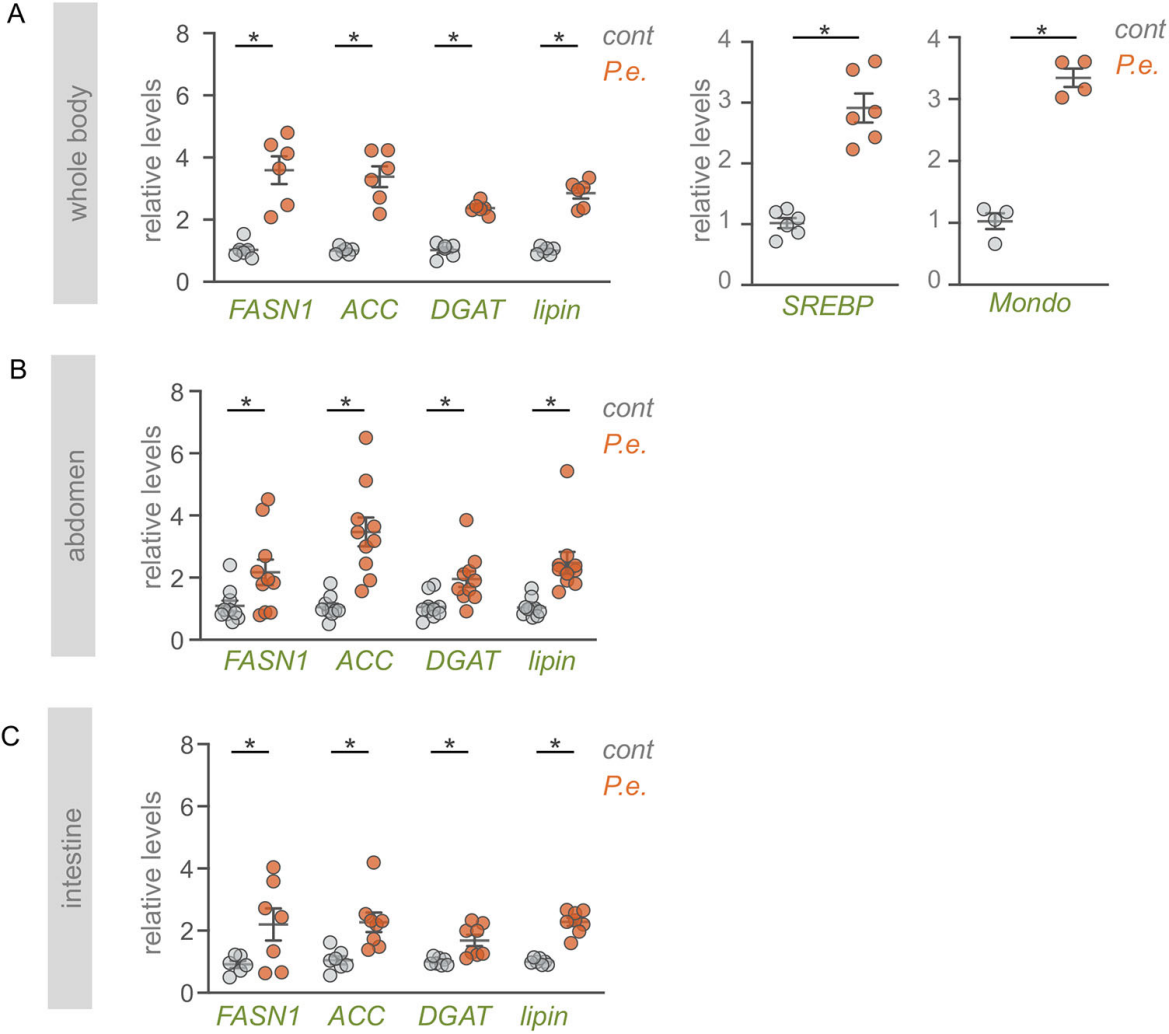
**Enteric infection leads to increased expression of lipid synthesis genes in the intestine and abdominal fat.** (A) qPCR analysis from whole-body samples of control or *P. entomophila* (*P.e.*)-infected flies of lipid synthesis genes (*FASN1*, *ACC*, *DGAT* and *Lipin*) and two transcription factors (SREBP and Mondo) that control the expression of lipid synthesis genes. (B) qPCR analysis of lipid synthesis genes from abdominal samples of control or *P. entomophila*-infected flies. (C) qPCR analysis of lipid synthesis genes from intestinal samples of control or *P. entomophila*-infected flies. Bars represent mean±s.e.m., individual data points are plotted as circles. *P<0.05, two-tailed unpaired Student's *t*-test.

**Fig. 6.**
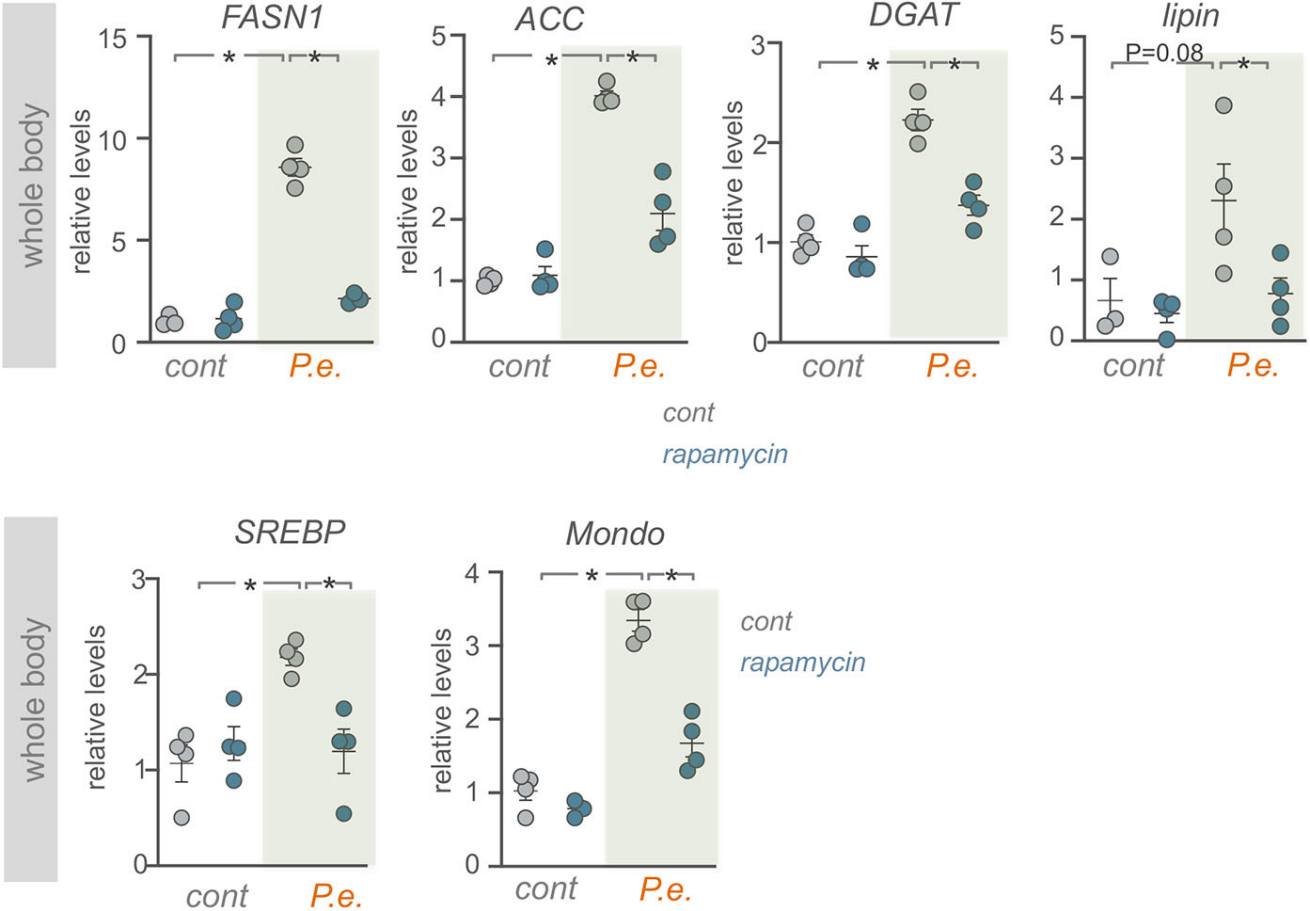
**TOR is required for the increased expression of lipid synthesis genes induced by enteric infection.** qPCR analysis of lipid synthesis genes and the transcription factors SREBP and Mondo in *w^1118^* flies pre-treated for 24 h with either DMSO (control, grey symbols) or rapamycin (blue symbols), followed by 24 h of either sucrose (control) or 24 h oral *P. entomophila* (*P.e.*) feeding. Bars represent mean±s.e.m., individual data points are plotted as circles. *P<0.05, two-way ANOVA followed by two-tailed unpaired Student's *t*-test.

Our results suggest that TOR is needed to limit excessive loss of lipid stores post infection. To do this, TOR may be blocking excess lipase function (to limit lipolysis) or may be increasing lipid synthesis (to resupply new lipids following infection). To explore this further we examined the expression of genes required for *de novo* lipid synthesis. We found that flies infected orally with *P. entomophila* showed a significant upregulation in mRNA expression levels of genes required for lipid synthesis such as *Acetyl-CoA carboxylase* (*ACC*), *Fatty acid synthase 1* (*FASN1*), *midway* (*mdy*; DGAT) and *Lipin* (Fig. 5A). Moreover, the expression levels of two transcription factors, SREBP and Mondo, which promote the transcription of these genes (Heier and Kuhnlein, 2018; Mattila and Hietakangas, 2017) were also upregulated (Fig. 5A). In *Drosophila* adults, lipid synthesis occurs in the intestine, the fat body and oenocytes (Heier and Kuhnlein, 2018). We therefore carried out tissue-specific analysis of lipid synthesis gene expression in both intestines and isolated abdominal samples (which are enriched in fat body and oenocytes). In both cases we saw that enteric *P. entomophila* infection increased expression levels of lipid synthesis genes, suggesting that following infection new lipid synthesis is induced in several important lipid metabolic tissues (Fig. 5B,C). To explore how TOR might be involved in these effects, we examined the effects of rapamycin feeding to inhibit TOR. We found that the *P. entomophila*-induced increase in expression of lipid synthesis genes and transcription factors was significantly blunted in flies that had been fed rapamycin to block TOR (Fig. 6). These results suggest that one role for the increased TOR activity that we see upon infection may be to induce *de novo* lipid synthesis to replenish the lipid stores that are mobilized following enteric infection.

### Infection promotes glycogen mobilization through TOR signalling

**Fig. 7.**
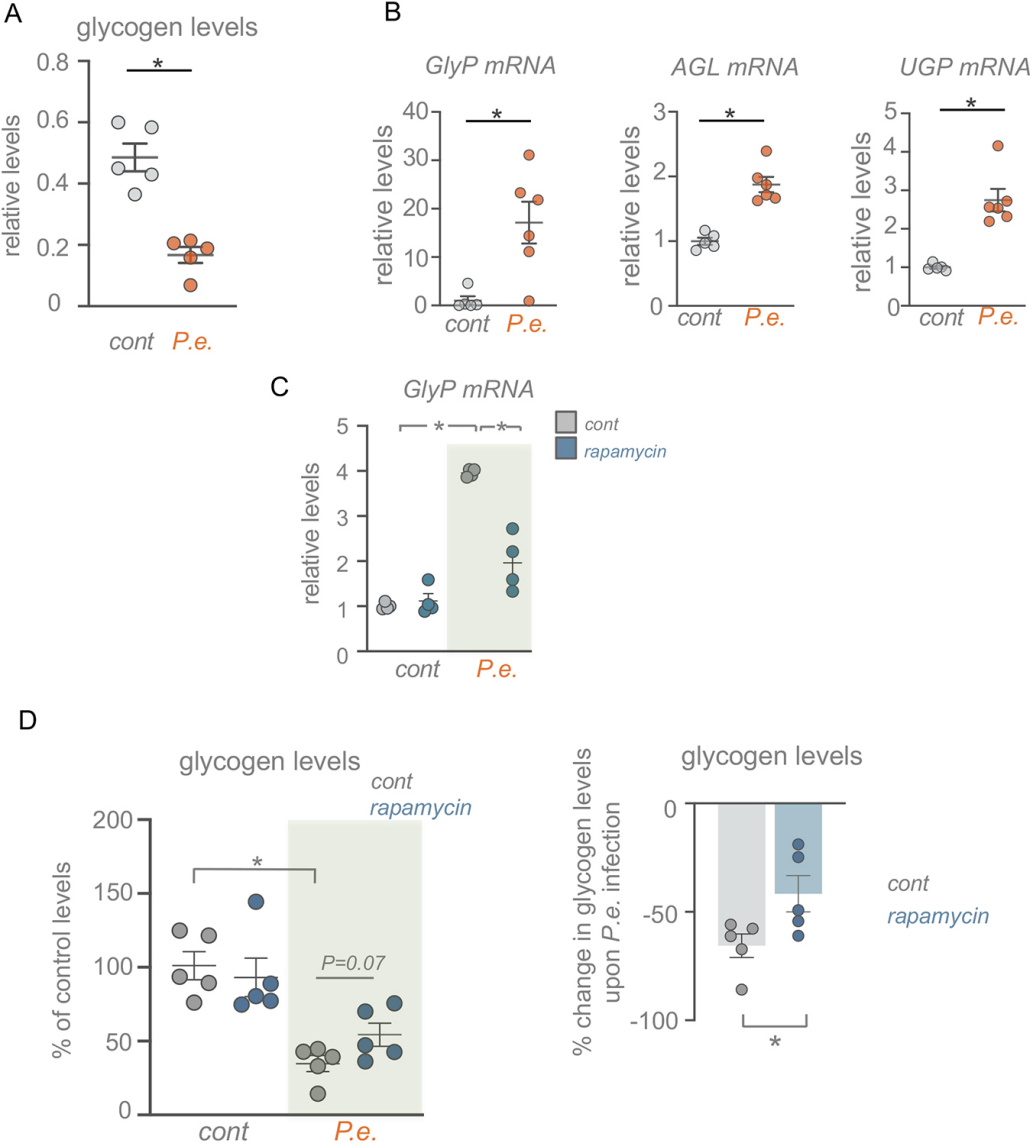
**Enteric infection leads to glycogen mobilization in part through TOR activity.** (A) Total glycogen levels in control versus 24 h *P. entomophila* (*P.e.*)-infected adults. (B) qPCR analysis of glycogen breakdown genes from whole-body samples of control or 24 h *P. entomophila*-infected flies. (C) *w^1118^* mated females were pre-treated for 24 h with either DMSO (control) or rapamycin, followed by 24 h of either sucrose (control) or 24 h oral *P. entomophila* feeding (grey bars). Whole animals were then processed for qRT-PCR analysis of GlyP mRNA. (D) *w^1118^* mated females were pre-treated for 24 h with either DMSO (control) or rapamycin, followed by 24 h of either sucrose or 24 h oral *P. entomophila* feeding. Whole animals were then processed for measurement of total glycogen assays. Bars represent mean±s.e.m., individual data points are plotted as circles. *P<0.05, two-tailed unpaired Student's *t*-test (A,B) or two-way ANOVA followed by a two-tailed unpaired Student's *t*-test (C,D).

*De novo* lipid synthesis often relies on metabolic conversion of glucose into acetyl-CoA which can then serve as the source for new TAG synthesis (Heier and Kuhnlein, 2018). Flies can acquire glucose both from their diet and also from mobilization of stored glucose in the form of glycogen (Mattila and Hietakangas, 2017). When we infected flies with *P. entomophila* we saw a significant decrease in whole-body glycogen levels, as has been reported following systemic infection (Chambers et al., 2012; Dionne et al., 2006), and an increase in mRNA expression levels of several genes required for glycogen mobilization such as *Glycogen phosphorylase* (*GlyP*), *CG9485* (orthologous to human *AGL*), and *UDP-glucose pyrophosphorylase* (*UGP*) (Fig. 7A,B). These results indicate that infection triggers a mobilization of host glycogen stores. Interestingly, when we blocked TOR signalling by feeding flies rapamycin, this prevented the infection-induced increase in mRNA levels of *GlyP*, the limiting enzyme for glycogen mobilization (Fig. 7C). Moreover, we saw that infection-mediated mobilization of glycogen was reduced in rapamycin-fed animals (Fig. 7D). Taken together, these results suggest that infection leads to depletion of stored glycogen in part through TOR signalling. Thus, one possibility is that this TOR-dependent mobilization of glycogen is used to fuel tissue metabolism during infection and also may provide glucose for TOR-induced *de novo* synthesis of TAGs.

## DISCUSSION

In this paper we show that enteric infection with Gram-negative bacteria leads to both a local (intestinal) and systemic increase in TOR signalling. We found that this induction is required to replenish mobilized lipid stores and is needed for optimal survival following infection. We found that the induction of TOR occurred independently of either IMD or ROS signalling, the two best-studied pathways induced upon enteric infection with Gram-negative bacteria. Instead, our data suggest that enteric infection induces a direct stimulation of TOR in the intestine and also a non-autonomous induction of systemic insulin/PI3K signalling.

It is interesting to compare our results with previous work examining enteric infection and TOR signalling. One study showed that oral infection with the Gram-negative bacteria *E. carotovora carotovora* induced a rapid induction of intestinal TOR activity, as measured by phosphorylation of the TOR effector 4EBP, and that this induction was specifically seen in the intestinal stem cells (Haller et al., 2017). We saw a similar induction of TOR in stem cells, but we observed that it also occurred in the large enterocytes, the main absorptive, barrier and metabolic cells of the intestine. In contrast to both this study and our work, another paper showed that *P. entomophila* infection actually led to a reduction in TOR and a decrease in protein synthesis (Chakrabarti et al., 2012). We found that the induction of TOR signalling was accompanied by an increase in levels of tRNAs, pre-RNA and ribosome synthesis genes, each of which are known to be targets of TOR signalling (Grewal et al., 2007; Li et al., 2010; Marshall et al., 2012), suggesting an increase in protein synthesis. The reasons for each of these differences in TOR responses to enteric infection could be due to differences in either the pathogenicity or levels of the enteric bacteria. Alternatively, they may reflect differences in fly diet and/or commensal bacterial between each study. Indeed, cross-talk between commensal and pathogenic bacteria, as well as interactions with dietary nutrients, have been shown to impact intestinal physiology and gut epithelial responses (McCarville et al., 2020). These effects are often mediated through bacterial-derived small molecules or metabolites. Given that intracellular TOR signalling can be stimulated by extracellular small molecules such as amino acids, nucleotides and sugars (Ben-Sahra and Manning, 2017; Valvezan and Manning, 2019), it is possible that these mechanisms may explain the induction of intestinal TOR that we observed.

Our data suggest that the systemic induction of TOR may rely on insulin/PI3K signalling. Thus, we saw that enteric infection increased Akt phosphorylation and altered expression of FOXO targets in whole animals and remote tissues such as the abdominal fat tissues. The insulin pathway is one of the main endocrine regulators of metabolism in flies and extensive work has shown that one main way that it is stimulated is through increased expression of brain-derived ILPs in response to peripheral tissue-to-brain signalling (Grewal, 2012; Koyama et al., 2020). We saw increased expression of three ILPs, suggesting a potential role for gut-to-brain signalling. Indeed, some of the gut-derived peptides in flies have previously been shown to control brain ILP expression (Alfa et al., 2015; Ren et al., 2015; Sano et al., 2015; Yoshinari et al., 2021) and infection has been shown to stimulate the enteroendocrine cells that produce these peptides (Park et al., 2016). Interestingly, our induction of systemic insulin with *P. entomophila* contrasts with studies showing other bacterial pathogens such as *V. cholera* and *Mycobacterium marinum* can suppress insulin/PI3K signalling in flies (Dionne et al., 2006; Hang et al., 2014), suggesting that the type of pathogenic bacteria is an important determinant of host physiological responses.

We found that the stimulation of TOR signalling by *P. entomophila* infection was not required for induction of the AMPs, the main anti-bacterial resistance response in flies, and had no effect on pathogen burden. The AMPs are primarily induced by IMD/Relish signalling following enteric infection. Interestingly we saw that infection survival was reduced only when we simultaneously blocked both IMD signalling (*Relish* mutants) and TOR signalling (rapamycin feeding). Based on these data, one simple model is that, upon infection, the IMD pathway is induced to initiate resistance (antibacterial defences), whereas TOR induction plays a role in tolerance responses (adaption to pathogen infection). Another possibility, is that the effects of TOR induction become important when the IMD pathway is compromised, hence leading to exacerbated death in *Relish* mutants treated with rapamycin.

Tolerance responses are defined as alterations in host biology that limit pathology and promote survival without affecting pathogen load (Ayres and Schneider, 2012; Medzhitov et al., 2012). These can involve adaptations in host metabolism (Cumnock et al., 2018), changes in host behaviour or induction of host tissue protective and repair processes (Martins et al., 2019). It is becoming clear that these responses are as important as resistance (anti-pathogenic) responses in determining host survival upon infection, and, as a result, there is increasing interest in determining mechanisms that control tolerance. In the context of enteric infection, recent studies have emphasized how gut-mediated changes in whole-body level physiological programmes such as systemic insulin signalling and glucose and lipid metabolism play an important role in tolerance responses (Sanchez et al., 2018; Schieber et al., 2015). We saw that enteric infection led to a transient reduction in total TAGs and a decrease in intestinal and fat body lipid stores. These results are consistent with previous reports that also described how both enteric and systemic infection with pathogenic bacteria such as *M. marinum* and *V. cholera* can reduce both intestinal and fat body lipid levels (Dionne et al., 2006; Hang et al., 2014; Kamareddine et al., 2018a,b; Zhao and Karpac, 2021). Constitutive activation of the IMD pathway in the *Drosophila* fat body can promote lipid mobilization (Davoodi et al., 2019), suggesting that lipid loss may be a general feature of infection with pathogenic bacteria in flies. One likely possibility is that this lipid loss reflects mobilization of lipid stores to fuel energetically costly host immune responses perhaps through a switch to fatty acid oxidation, which is a type of metabolic reprogramming often seen upon infection (Cumnock et al., 2018). We saw that infection increased the expression of lipoproteins that are needed for transport of fat body- and gut-derived lipids to other tissues. Moreover, a recent study described inter-individual differences in mobilization and transport of lipids via these lipoproteins as a key determinant of infection susceptibility (Zhao and Karpac, 2021).

In the context of infection-mediated lipid mobilization, we saw that one function for TOR appeared to be limiting excess lipid loss. Thus, when we rapamycin-treated flies we saw that the transient decrease in lipid stores following infection developed into a progressive loss of lipid stores. Our results suggest that TOR functions to prevent excess lipid loss by promoting *de novo* lipid synthesis through increased expression of lipid synthesis genes and two transcription factors, Mondo and SREBP, that control the expression of these genes (Heier and Kuhnlein, 2018; Mattila and Hietakangas, 2017). These lipid synthesis genes are enriched for expression in the fat body, oenocyte and the intestine (Leader et al., 2018) and we saw increased expression in both intestinal and isolated abdominal samples (that are enriched in fat body and oenocytes). We therefore propose that enteric pathogens can, through direct effects on the gut and indirect effects on systemic insulin, increase TOR activity in the gut and the abdominal adipose tissues (fat body/oenocytes) to stimulate lipid synthesis gene expression in these organs. It is also possible that TOR regulation of lipid metabolism in one tissue may non-autonomously mediate effects on lipid metabolism in other tissues as has been previously described (Kamareddine et al., 2018a; Song et al., 2014; Zhao and Karpac, 2017, 2020). Future studies using tissue-specific genetic modulation may help pin-point each of the specific site(s) of TOR action. We also found that TOR signalling regulated glycogen mobilization following infection: we saw that rapamycin blocked the induction of glycogen phosphate, which is required to mobilize glycogen, and we saw that the infection-mediated loss of glycogen was partially prevented by rapamycin. Together, our data suggest that, upon infection, TOR is required to limit lipid loss but is needed for proper glycogen mobilization, which may be used to fuel immune metabolic responses, and perhaps also to supply the glucose needed for *de novo* lipid synthesis.

A central theme of our work is that alterations in host lipid metabolism are an important component of immune responses. This is supported by previous studies in flies that have described how both intestinal and fat body lipid metabolism are needed for effective immune responses (Chakrabarti et al., 2014; Harsh et al., 2019; Kamareddine et al., 2018a; Lee et al., 2018; Martinez et al., 2020). Our work pinpoints TOR as a central modulator of enteric infection-mediated changes in lipid metabolism, likely as a mechanism of infection tolerance. The intestine also plays a central role coordinating other aspects of fly physiology such as repair of local tissue damage (Colombani and Andersen, 2020) and modulation of feeding behaviour (Hadjieconomou et al., 2020; Miguel-Aliaga et al., 2018; Redhai et al., 2020). Given previous work implicating these processes as regulators of infection tolerance (Ayres and Schneider, 2012; Rao et al., 2017), our finding that TOR is induced in the gut suggests it may also play a role in these other important responses to infection.

## MATERIALS AND METHODS

### *Drosophila* stocks and culturing

Flies were kept on medium containing 150 g agar, 1600 g cornmeal, 770 g Torula yeast, 675 g sucrose, 2340 g D-glucose, 240 ml acid mixture (propionic acid/phosphoric acid) per 34 l water and maintained at 25°C. The following lines were used in this study (Bloomington Drosophila Stock Center stock numbers indicated): *w^1118^*, *imd^[|EY08573]^* (17474), *rel^E20]^* (9457), *rel^E38]^* (9458), *UAS-ImpL2* (Sarraf-Zadeh et al., 2013), *da-geneswitch-Gal4* (Sun et al., 2014)*.* Induction of gene expression using the GeneSwitch system was carried out by feeding flies RU486 (100 µM) for 3 days.

### Enteric infections

Enteric infections were performed using previously described methods (Buchon et al., 2010; Zhao and Karpac, 2021). Briefly, *P. entomophila* from overnight cultures were pelleted and resuspended in 5% sucrose solution (in sterile PBS) such that the final concentration of bacteria was OD_600_=200, except for the experiment described in Fig. S4C where concentrations from OD_600_=1-200 were tested. To prepare infection vials, bacterial pellets were dissolved in filter sterilized 5% sucrose/PBS. Chromatography paper (Fisher) discs were dipped in the bacterial solution (5% sucrose was used as a control) and were carefully placed on standard fly food vials such that they covered the entire food surface. Adult females were first subjected to a 2 h starvation in empty vials at 29°C. Then 10-12 flies were transferred to each infection vial and placed in a 29°C incubator for the duration of the assay.

### Adult survival assay

Adult female flies were infected as above. Post infection, the flies were transferred to fresh food vials every 2 days. The number of deaths was scored every 24 h.

### Rapamycin and chemical feeding

For treatment with rapamycin, 3- to 5-day-old female flies were shifted on vials containing 100 µM rapamycin dissolved in standard *Drosophila* food for 24 h. DMSO dissolved in food was used as a control. After 24 h of rapamycin pre-treatment, the flies were then transferred to infection vials mixed with 100 µM final concentration of rapamycin or DMSO. Chemical intestine stressors [25 µg/ml bleomycin (Sigma-Aldrich, 9041-93-4), 5% DSS (Sigma-Aldrich, 9011-18-1), 2 mM paraquat (Sigma-Aldrich, 75365-73-0)] were used to induce intestine-specific stress in *w^1118^* flies. To block ROS, flies were fed the antioxidant N-acetyl cysteine at a concentration of 100 mM. Uracil was fed at a concentration of 20 nM. A 5% sucrose solution was used as a solvent for all the mentioned chemicals, and a 5% sucrose solution alone was used as the control for all experiments. We used 500 µl of each solution to completely soak a piece of 2.5 cm×3.75 cm chromatography paper (Fisher), which was then placed inside an empty vial, and 5- to 7-day-old mated females (*n*=10-12/vial) were then added to the vials.

### Bacterial load measurements

Adult flies were surface sterilized by washing in 70% ethanol and then sterile water. Groups of five flies were then homogenized using a sterile pestle. The homogenate was then serially diluted, and the serial dilutions were plated onto LB plates and incubated overnight at 29°C. The number of separate, well-defined colonies on each plate were then counted. The number of CFU/fly for each plate was calculated using the following formula: CFU/fly=[(colony number)×(dilution factor)/plating volume]×total volume of initial homogenate/5 (number of flies per condition).

### SDS-PAGE and western blotting

Intestines (10 per sample) were dissected in ice cold 1× PBS and immediately lysed in ice-cold lysis buffer containing 20 mM Tris-HCl (pH 8.0), 137 mM NaCl, 1 mM EDTA, 25% glycerol, 1% NP-40, 50 mM NaF, 1 mM PMSF, 1 mM DTT, 5 mM sodium orthovanadate (Na_3_VO_4_) and Protease Inhibitor cocktail (Roche, 04693124001) and phosphatase inhibitor (Roche, 04906845001). Protein concentrations were measured using the Bio-Rad Dc Protein Assay kit II (5000112). Protein lysates (15 µg to 30 µg) were resolved by SDS-PAGE and transferred to a nitrocellulose membrane, and then subjected to western blotting with specific primary antibodies and HRP-conjugated secondary antibodies, and then visualized by chemiluminescence (enhanced ECL solution; Perkin Elmer). Primary antibodies used in this study were: anti-phospho-S6K-Thr398 (1:1000, Cell Signaling Technology, 9209), anti-pERK T980 (1:1000, Cell Signaling Technology, 3179), anti-pAkt-S505 (1:1000, Cell Signaling Technology, 4054), anti-phospho S6 (1:500, gift from Aurelio Teleman, German Cancer Research Center, Heidelberg, Germany) and anti-actin (1:1000, Santa Cruz Biotechnology, sc-8432). Secondary antibodies were purchased from Santa Cruz Biotechnology (sc-2030, sc-2005, sc-2020 used at 1:10,000 dilution). Blots and band intensities were quantified using ImageJ.

### Phospho S6 immunostaining

Intestines were dissected in ice-cold 1× PBS and then fixed in 4% paraformaldehyde in 1× PBS (1:4 diluted from Pierce™ 16% formaldehyde, 28906) at room temperature (RT) for 30 min. Post fixation, the tissues were washed with 1× PBS+0.1% Triton X-100 for 10 min. The tissues were then blocked in 1× PAT buffer+2% foetal bovine serum (FBS) for 2 h at RT. The tissues were then transferred to fresh PAT containing the primary antibody overnight at 4°C. The primary antibody (anti-phospho S6, 1:200) incubation was followed by three washes at RT with 1× PBT+2% FBS for 30 min each. The tissues were then incubated with secondary antibody in PBT without the serum at RT for 2 h, followed by 3×30 min washes in PBT without serum at RT. Finally, the tissues were incubated with 1:10,000 of Hoechst 33342 (Invitrogen) in PBT to stain the nuclei. The tissues were then mounted on glass slides with coverslips using Vectashield (Vector Laboratories). The slides were visualized under a Zeiss Observer Z1 microscope using the 10× and 20× objectives and with Zen-Axiovision software. When analyzing and capturing images, exposure levels were kept constant across all conditions and samples analyzed. At least 10 independent tissue samples were profiled in each experiment and representative images are shown in the figures.

### qRT-PCR

Total RNA was extracted from groups of five adults or 10 intestines using TRIzol reagent according to the manufacturer's instructions (Invitrogen, 15596-018). The RNA samples were treated with DNase (Ambion, 2238G) and then reverse transcribed using Superscript II (Invitrogen, 100004925). The cDNAs were then used as a template for subsequent qRT-PCRs using SyBr Green PCR mix (Thermo Fisher Scientific) and an ABI 7500 real time PCR system. The PCR data were normalized to actin mRNA or 5S rRNA levels. The primers used in this study can be found in Table S1.

### BODIPY staining

The adult intestines were dissected in ice cold 1× PBS. The tissue samples were then fixed in 4% paraformaldehyde in 1× PBS (1:4 diluted from Pierce™ 16% formaldehyde) at room temperature for 30 min. The fixation was followed by 2× washes for 5 min at RT with PBS. The BODIPY (Invitrogen) was diluted in PBS (1:100) and tissues were incubated in this solution for 30 min at RT. The samples were then washed twice with PBS for 10 min. Finally, the tissues were incubated with 1:10,000 Hoechst 33342 (Invitrogen) in PBT to stain the nuclei, followed by another wash with PBS for 10 min at RT. The tissues were then mounted on slides and visualized as mentioned above. At least 10 independent samples were profiled for each condition. Representative images were shown in the figures.

### Oil Red O staining

The adult intestines were dissected in ice-cold PBS. The tissue samples were then fixed in 4% paraformaldehyde in PBS at RT for 30 min. The fixation was followed by 2× washes for 5 min at RT with PBS. The intestines were then incubated in fresh Oil Red O (Sigma-Aldrich, O0625) solution. The solution was prepared by adding 6 ml of 0.1% Oil Red O in isopropanol and 4 ml ultra-pure dH_2_O, passed through a 0.45 µm syringe, followed by rinsing with distilled water. The tissues were mounted on a glass slide and imaged using a Zeiss Discovery V8 stereomicroscope. At least 10 independent samples were profiled for each condition. Representative images were shown in the figures.

### TAG and glycogen assays

The metabolic assays were performed as previously described (Tennessen et al., 2014). Briefly, animals were lysed and lysates were heated at 70°C for 10 min. Then they were incubated first with triglyceride reagent (Sigma-Aldrich, T2449) and then mixed with free glycerol reagent (Sigma-Aldrich, F6428). Colorimetric measurements were then made using absorbance at 540 nm and TAG levels calculated by comparing with a glycerol standard curve. Glycogen assays were performed by lysing animals in PBS and then heating lysates at 70°C for 10 min. For each experimental sample, duplicate samples were either treated with amyloglucosidase (Sigma-Aldrich, A1602) to break down glycogen into glucose, or left untreated, and then levels of glucose in both duplicates measured by colorimetric assay following the addition of a glucose oxidase reagent (Sigma-Aldrich, GAGO-20). Levels of glycogen in each experimental sample were then calculated by subtracting the glucose measurements of the untreated duplicate from the amyloglucosidase-treated sample. All experimental metabolite concentrations were calculated by comparison with glycogen and glucose standard curves.

### Statistical tests

qRT-PCR and metabolite data were analyzed by two-way ANOVA and/or Student's two-tailed unpaired *t*-test where appropriate. Survival data were analyzed using the Log-Rank test. Statistical tests were performed using GraphPad Prism software.

## Supplementary Material

Supplementary information
