# Supplementary material for: TOR signalling is required for host lipid metabolic remodelling and survival following enteric infection in Drosophila
Source: Dis Model Mech. 2022 May 9;15(5):dmm049551. doi: 10.1242/dmm.049551 (PMC9118046; doi:10.1242/dmm.049551)
Supplement: Supplementary information [file dmm-15-049551-s1.pdf]

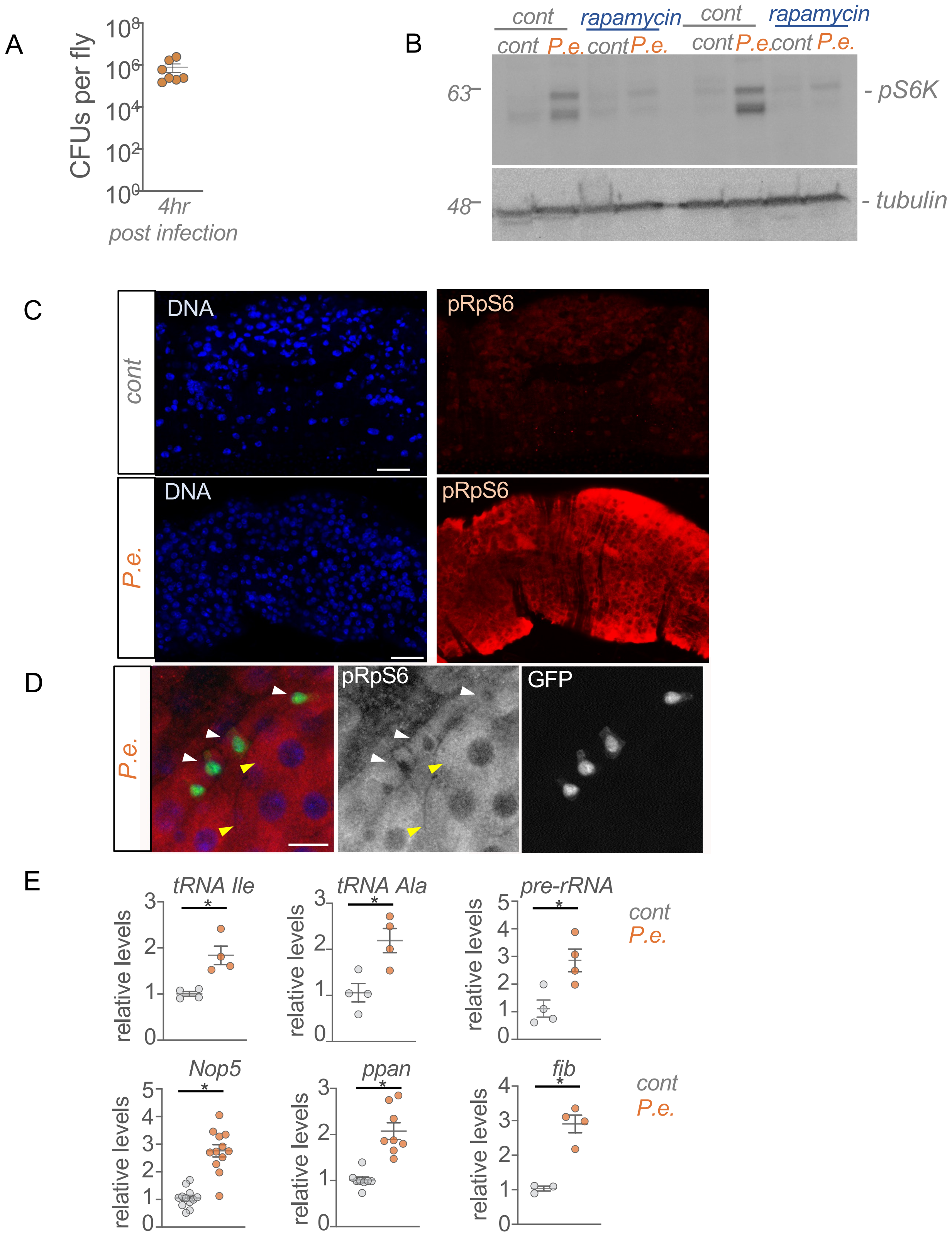

Fig. S1 (related to Figure 1). Infection with *P.e.* stimulates TOR activity in adult intestines. A) Pathogen abundance (in colony forming units, CFUs, per fly) at 4h following enteric *P.e.* infection. B) w^{1118} females were pretreated for 24 hours with either DMSO (cont) or rapamycin, followed by 4hr of either sucrose (cont) or 4hr *P.e.* feeding (grey bars). Intestinal samples were analyzed by western blot using antibodies to phosphorylated S6K and actin (as a loading control). C) Phosphorylated RpS6 (pRpS6) immunostaining in control vs 4hr *P.e.* infected flies. Blue, DNA; Red, anti-phospho RpS6 staining. Scale bar = 50 micrometres. Increased phospho S6 levels were observed in 10 out of 10 infected guts analyzed. D) Phosphorylated RpS6 (pRpS6) immunostaining in control vs 4hr *P.e.* infected flies. Blue, DNA; Red, anti-phospho RpS6 staining; Green, GFP positive ISC/EB cells marked by *esgGal4, UAS-GFP* (white arrowheads). Yellow arrowheads indicate large polyploid enterocytes. Scale bar = 10 micrometres. E) qPCR analysis of tRNA, pre-rRNA and ribosome synthesis genes from intestinal samples of control or 4hr *P.e.* infected flies. Bars represent mean +/-SEM, individual data points are plotted as circles. *p<0.05, Students t-test.

Disease Models & Mechanisms • Supplementary information

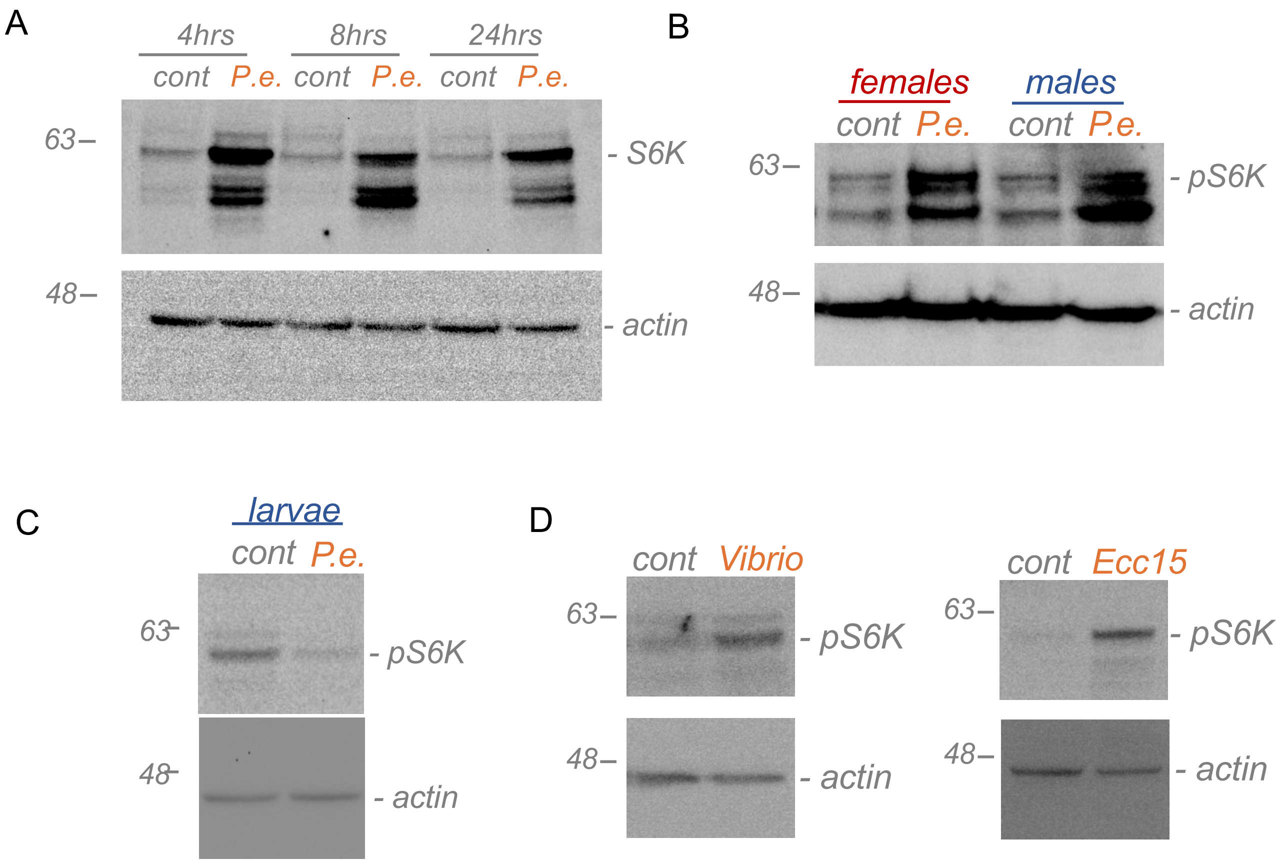

Fig. S2 (related to Figure 1). Enteric bacterial infection stimulates TOR activity in adult male and female intestines. A) Time course of *P.e.* infection on phosphorylated-S6K levels in intestines of w^{1118} mated females. Dissected intestines were analysed by western blotting using antibodies against phosphorylated-S6K and actin (loading control). B) Western blots of intestines from adult male and female flies subjected to 4hr oral *P.e* infection. Antibodies were against phosphorylated S6K (pS6K) and actin (loading control). C) Western blots of intestines from third instar larvae subjected to 4hr oral *P.e* infection. Antibodies were against phosphorylated S6K (pS6K) and actin (loading control). D) Western blots of intestines from adult flies subjected to 4hr oral infection with *V. Cholera* or *Ecc15*. Antibodies were against phosphorylated S6K (pS6K) and actin (loading control).

Disease Models & Mechanisms • Supplementary information

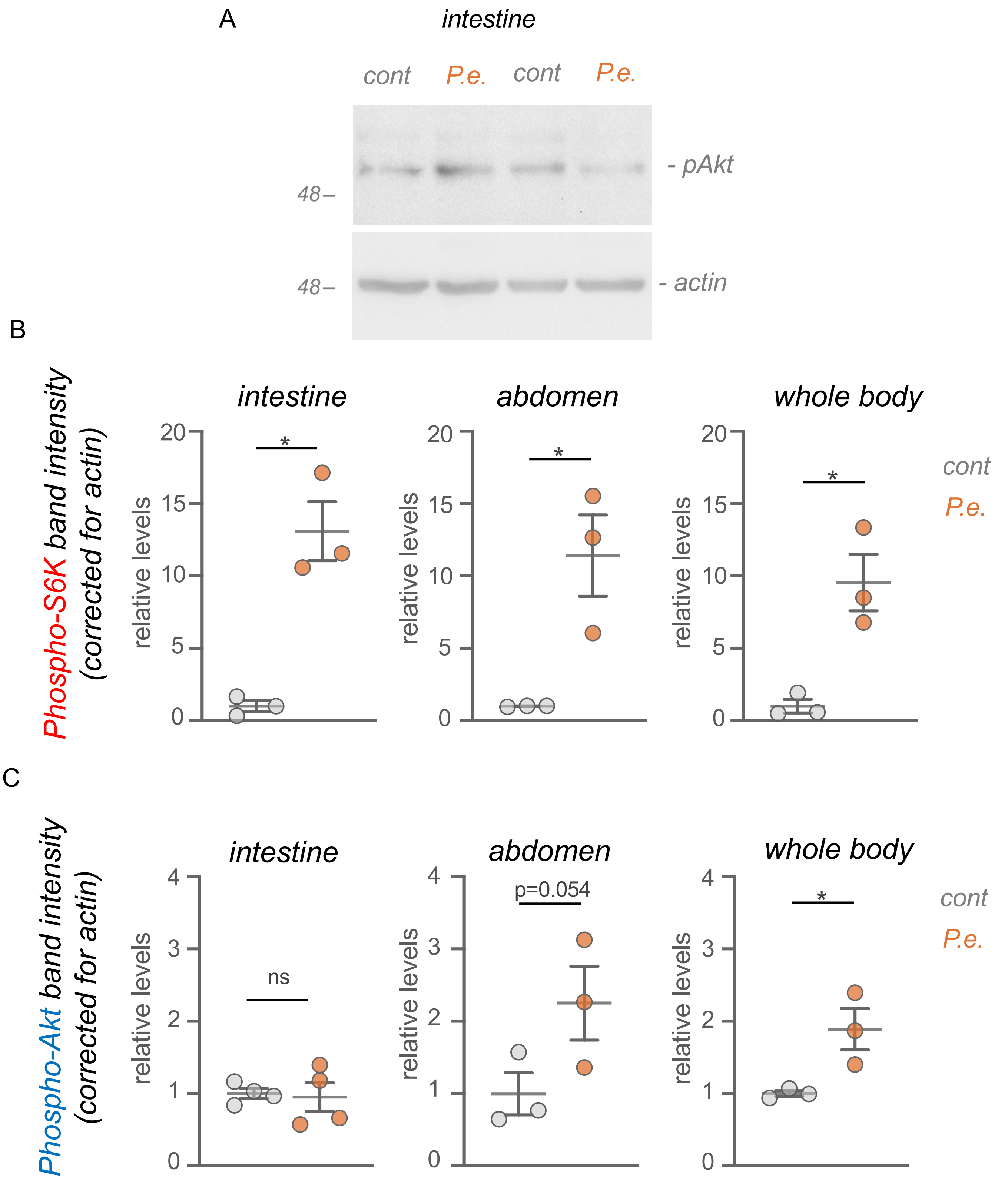

Fig. S3 (related to Figure 1). Enteric bacterial infection effects on S6K and Akt phosphorylation in the intestine, abdomen and whole-body. A) Western blots of intestinal samples from adult flies subjected to 4hr oral *P.e* infection using antibodies to phosphorylated Akt, (pAkt) and actin (as a loading control). B, C) Quantification of B) phosphoS6K and C) phosphoAkt, western blots of intestinal, abdominal and whole-body samples from adult flies subjected to 4hr oral *P.e* infection. Band intensities were corrected for actin levels. Bars represent mean +/-SEM, individual data points are plotted as circles. *p<0.05, ns= not significant, Students t-test.

Disease Models & Mechanisms • Supplementary information

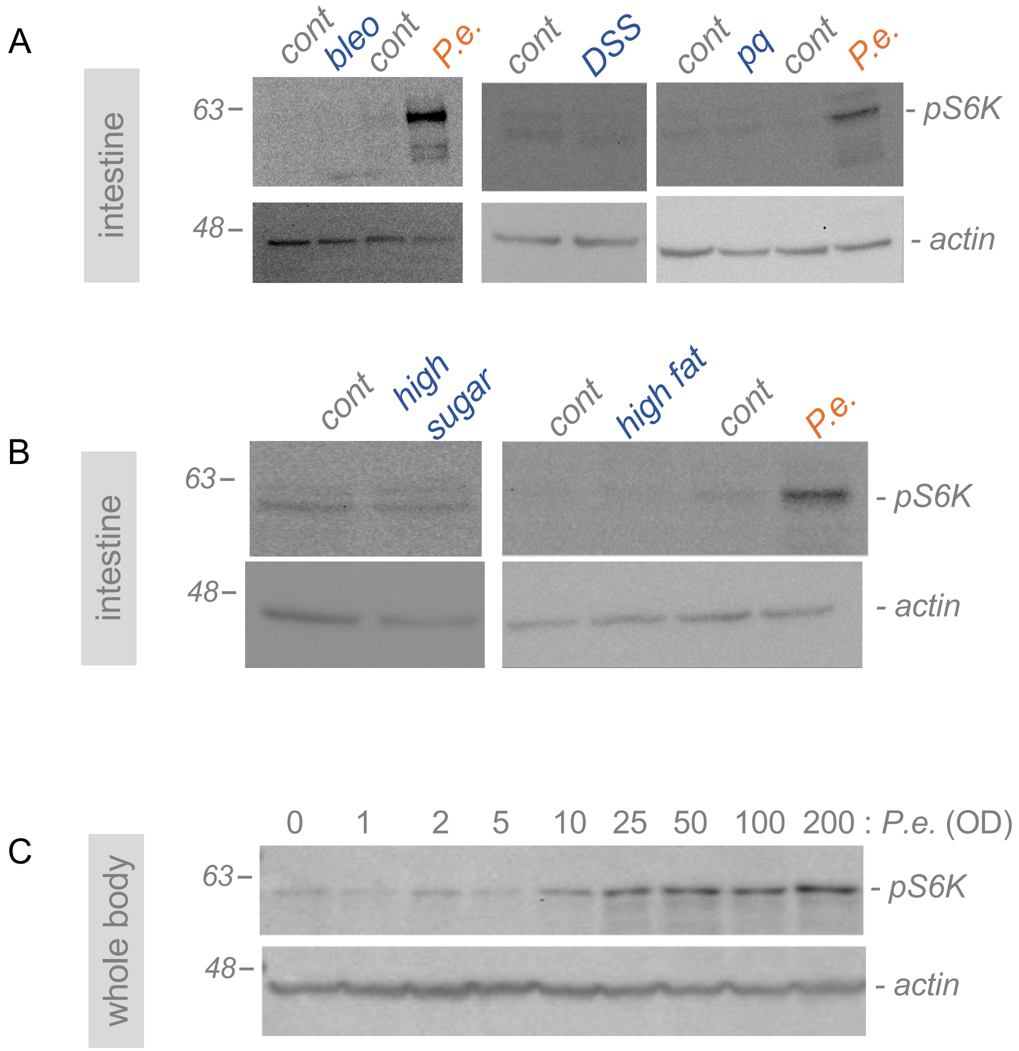

Fig. S4 (related to Figure 1). Enteric bacterial infection, but not other environmental stress, stimulates TOR activity in the intestine. A) Western blot of dissected intestines from adult flies subjected to 4hr treatments of chemical stressors: 25µg/ml Bleomycin, 5% DSS, 2mM paraquat. Antibodies were against phosphorylated S6K (pS6K) and actin (loading control). B) Western blot of dissected intestines from adult flies subjected to 4hr feeding with high sugar (40% sucrose), high fat (30% lard) or *P.e.* Antibodies were against phosphorylated S6K (pS6K) and actin (loading control). C) Western blot of whole-body samples from adult flies subjected to 4hr feeding with different concentrations (OD_{600} 1-200) of *P.e.* Antibodies were against phosphorylated S6K (pS6K) and actin (loading control).

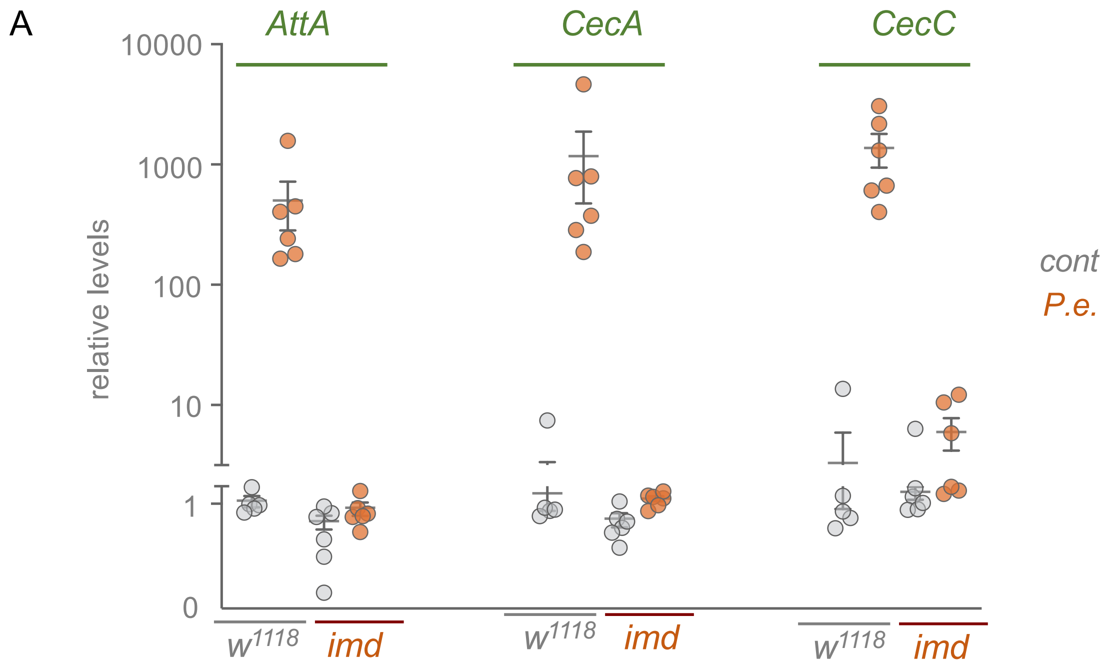

Fig. S5 (related to Figure 2). AMP induction is blocked in *imd* mutants. qPCR analysis of AMP genes from whole-body samples of w^{1118} or *imd* mutant animals following 24hr of *P.e.* infection. Bars represent mean +/-SEM, individual data points are plotted as circles.

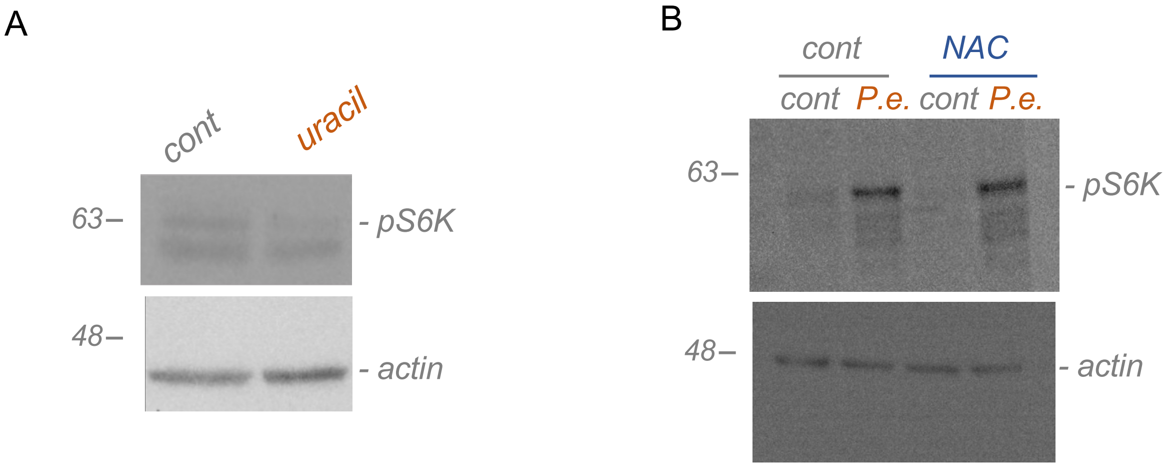

Fig. S6 (related to Figure 2). Uracil or ROS are not involved in TOR induction. A) Western blot of dissected intestines from adult control vs 4hr uracil-fed flies. Antibodies were against phosphorylated S6K (pS6K) and actin (loading control). B) Western blot of dissected intestines from adult control vs 4hr *P.e.* infected flies that had been pretreated for 2 days with either water (control) or N-acetyl cysteine (NAC). Antibodies were against phosphorylated S6K (pS6K) and actin (loading control).

Disease Models & Mechanisms • Supplementary information

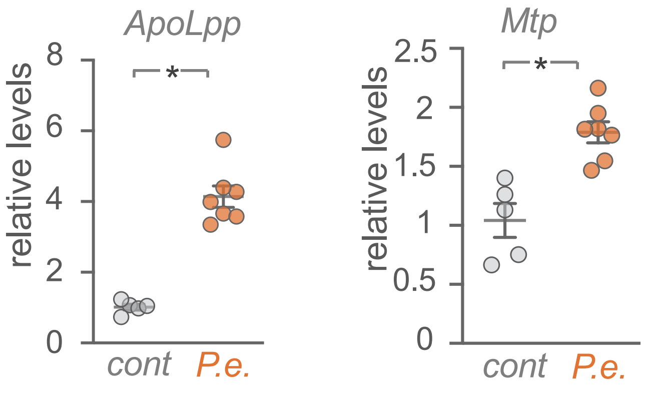

Fig. S7 (related to Figure 4). Enteric infection increases the expression of apolipoprotein mRNAs. qPCR analysis of *ApoLpp* or *Mtp* from whole-body samples of *control vs* 24hr infected *P.e.* animals. Bars represent mean +/-SEM, individual data points are plotted as circles.

Disease Models & Mechanisms • Supplementary information

Table S1. A list of primers used in this study

Gene name	Forward sequence	Reverse sequence
Fatty acid synthetase 1(CG3523) (FASN1)	TCCCAGAGGCAAACATTACC	TCGGGGAAATGAAGAAGATG
Acetyl-CoA carboxylase (CG11198) (ACC)	GCCAAGAGCATAACGAGGAG	GCTCCAGATGCCGGTAAATA
Midway (DGAT)	CTCTTTAGTGCATATCTCGCTCTG	AACAAGCCCAAGCCCTCT
lipin	CTCGGCGGCTATCAAAA	ACCTTGTCGTTGTGCTTCCA
Lipid storage droplet (LSD)-2	AGAGCAAGGTGATCGATGTG	ACTCCGTTGACAGCCAGACT
Mondo	GCGGCGTTACAACATAAAGA	CTCCATGCGCAAAGCTTCAA
SREBP	AAGGACACTCTCTGGGCTGA	GCTTGATCCTGCCGTACAAT
GlyP	CAACTGGTTGCTCTGAAGAAGTG G	CTGGCGCTTGTACTCGTGAATACG
tRNA Ala	GCGGCCGCACTTTCACTGACCGGA A ACG	GCGGCCGCGCCCGTTCTAACTTTTT GGA
tRNA Ile	CGACCTTCGCGTTATTAGCA	GGCCCATTAGCTCAGTTGGT
tRNA Arg	GCGGCCGCGTCCGTCCACCAATG AA AAT	GCGGCCGCCGGCTAGCTCAGTCGG T AGA
Diptericin	GGCTTATCCGATGCCCGACG	TCTGTAGGTGTAGGTGCTTCCC
Attacin A	AGGAGGCCCATGCCAATTTA	CATTCCGCTGGAACTCGAAA
Cecropin A	TCTTCGTTTTCGTCGCTCTCA	ATTCCCAGTCCCTGGATTGTG

Disease Models & Mechanisms • Supplementary information

Cecropin C	TCATCCTGGCCATCAGCATT	CGCAATTCCCAGTCCTTGAAT
Drosocin	TTTGTCCACCACTCCAAGCAC	ATGGCAGCTTGAGTCAGGTGA
Metchnikowin	CGATTTTTCTGGCCCTGCT	CCGGTCTTGGTTGGTTAGGAT
Act5C	GAGCGCGGTTACTCTTTCAC	ACTTCTCCAACGAGGAGCTG
RpS9	AAACCTGCTCGGTTGAATTG	TTGTTGCGCAGACCATACTC
5S RNA	ACGACCATACCACGCTGAAT	AGCGGTCCCCCATCTAAGTA

Disease Models & Mechanisms • Supplementary information
